# Pilot scale isolation of exopolysaccharides from *Streptococcus thermophilus* DGCC7710: Impact of methodical details on macromolecular properties and technofunctionality

**DOI:** 10.1002/elsc.202000073

**Published:** 2020-12-09

**Authors:** Carsten Nachtigall, Georg Surber, Jannis Bulla, Harald Rohm, Doris Jaros

**Affiliations:** ^1^ Chair of Food Engineering Technische Universität Dresden Dresden Germany

**Keywords:** acid milk gels, emulsion, exopolysaccharides, isolation, lactic acid bacteria

## Abstract

Exopolysaccharides (EPS) from *Streptococcus thermophilus* provide similar technofunctionality such as water binding, viscosity enhancing and emulsifying effects as commercial thickeners at a significant lower concentration. Despite their high technofunctional potential, hetero polysaccharides from lactic acid bacteria are still not commercially used in unfermented foods, as the small amount of synthesised EPS calls for a high isolation effort. This study aims to analyse the macromolecular properties of EPS and cell containing isolates from *S. thermophilus* DGCC7710 obtained by different isolation protocols, and to link these data to the technofunctionality in model food systems. The EPS content of the isolates was affected by the microfiltration/ultrafiltration membranes used for cell removal/dialysis, respectively, and was 89% at maximum. There was no link between purity of the isolates, molecular mass (3 × 10^6^ Da) and intrinsic viscosity (0.53 – 0.59 mL/mg) of the EPS. After adding EPS containing isolates to milk, gel stiffness after acidification increased by 25% at maximum, depending on the type and concentration of the specific isolate. Partly purified, cell containing isolates were effective at low absolute EPS concentration (approx. 0.1 g/kg) and therefore represent, together with their simple isolation protocol, an interesting approach to introduce microbial EPS into non‐fermented products.

**Abbreviations**
cEPS: capsular exopolysaccharides
EPS: Exopolysaccharides
FD: freeze‐dried
fEPS: free exopolysaccharides
SD: spray‐dried
*S. thermophilus*: *Streptococcus thermophilus*
tEPS: total exopolysaccharides

## 1 INTRODUCTION

Polysaccharides are important additives for the adjustment of texture, rheology and stability of foods. Commercially important hydrocolloids are derived from plants (e.g. starch, pectin, guar gum) [1], algae (carrageenan) [2] or fungi (pullulan) [3]. Some authors raised, however, concerns about the safety of some of these thickeners, e.g. carrageenan [4, 5]. Being of microbial origin, only xanthan, a hetero exopolysaccharides (EPS) produced by *Xanthomonas campestris*, and the homo EPS dextran from *Leuconostoc mesenteroides* are currently permitted as food additives [6, 7]. Other microbial polysaccharides, especially hetero EPS from lactic acid bacteria with GRAS/QPS status (e.g. *Streptococcus thermophilus (S. thermophilus)* or *Lactococcus lactis*), are subject of current research [8, 9], but still not permitted as additive. To apply such EPS in non‐fermented products, it must be distinguished between EPS synthesis, isolation and application. As hetero EPS are produced only in low amounts (≤ 1.0 g/L) and synthesis is growth associated [10], suitable isolation and purification procedures are still a major challenge [11].

The extraction of EPS from fermentation media usually comprises the removal of cells (microfiltration, centrifugation), proteins (acid precipitation, enzymatic hydrolysis), monosaccharides, salts and other minor components (dialysis), and EPS precipitation (with acetone or ethanol) and drying (freeze drying, spray drying) [12]. The EPS content of the isolates, also referred to as purity, depends on cultivation parameters, details of the isolation procedure and EPS type, and ranges between 2 and 98% [13]. For example, C and N sources required for bacterial growth and EPS production that are present in MRS medium affect isolation, e.g. lactose enhances co‐precipitation of medium components with the EPS, resulting in lower purity [14]. Precipitation with trichloracetic acid and subsequent centrifugation for protein removal may also result in EPS co‐precipitation and thus a reduction in EPS yield of up to 50% [15]. EPS purity can be improved through the right choice and amount of organic solvent used for polysaccharide precipitation [16], and repeated precipitation further increases isolate purity [17]. Notararigo et al. [18] showed that the isolation procedure needs to be adapted for each strain, and neutral homo EPS are easier to isolate in a higher purity than hetero EPS.

**PRACTICAL APPLICATION**

Hetero exopolysaccharides (EPS) from lactic acid bacteria show a high potential for the use as thickening agents, as they exhibit similar technofunctional properties as commercial hydrocolloids, but already at significantly lower amount. However, the isolation of pure EPS is laborious and therefore not economically yet. To overcome this drawback, we applied simplified isolation protocols to a whey permeate medium fermented with *Streptococcus thermophilus* DGCC7710, resulting in isolates with different purities, macromolecular properties and technofunctionality in model food systems. We observed a positive effect on gel stiffness of chemically acidified milk for all isolates, and the increase in gel stiffness was determined by the absolute EPS amount added to milk. Partly purified, still cell containing isolates were already effective at low absolute EPS concentrations and seem, apart from the simplified isolation protocol, promising to introduce microbial EPS into non‐fermented products.

A number of studies demonstrated the high application potential of EPS in food systems. A positive correlation was found between the amount of EPS from *S. thermophilus* added to milk, and the stiffness of acid gels made thereof [19]. Compared to dextran, much lower concentrations were needed to induce comparable effects [20]. Similar experiments were performed with charged EPS [21]. Thickening properties in milk products correlated with macromolecular properties such as intrinsic viscosity or gyration radius [22, 23]. Other studies refer to emulsifying and stabilising properties of uncharged hetero EPS from *Acidobacterium* and *Bifidobacterium* species, alone or in combination with commercial hydrocolloids [24, 25]. In general, the described effects depended on various properties of the EPS such as molecular structure, monosaccharide composition or molecular mass, making the comparison of EPS still challenging [19, 26, 27].

In the present study, our standard EPS isolation procedure [28] was altered and simplified concerning the number of isolation steps, usage of chemicals, and need for time, and applied to one *S. thermophilus* strain. The aim was to link composition and macromolecular properties of the isolates, obtained by different procedures, to the resulting technofunctionality of the EPS in models for fermented and non‐fermented foods, namely milk gels and emulsions.

## 2 MATERIALS AND METHODS

### 2.1 Materials

*S. thermophilus* DGCC7710 was provided by Danisco Deutschland GmbH (Niebüll, Germany). Whey permeate powder was from Wheyco GmbH (Altentreptow, Germany), skim milk powder from Sachsenmilch Leppersdorf GmbH (Leppersdorf, Germany), and glucono‐δ‐lactone (GDL) from Kampffmeyer Nachf. GmbH (Ratzeburg, Germany). Canola oil was purchased in a local supermarket, tryptone and Tween^®^ 80 from Carl Roth GmbH & Co. KG (Karlsruhe, Germany), and all other chemicals from Merck KGaA (Darmstadt, Germany).

### 2.2 Exopolysaccharide production and isolation

DGCC7710 was cultivated in a 70 L bioreactor (Applikon^®^ Biotechnology BV, Delft, The Netherlands) anaerobically at 40°C and pH 6.0, using 60 g/L whey permeate powder in deionised water, enriched with 10 g/L tryptone, 2 g/L ammonium sulphate, 9 g/L glucose and 34.2 g/L lactose) [28].

**FIGURE 1:**
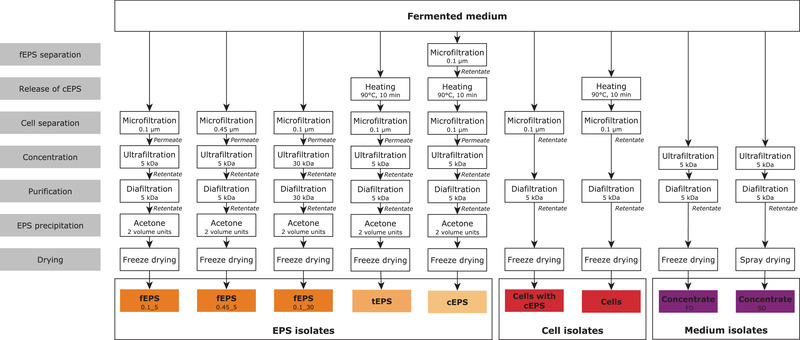
Procedures for obtaining different EPS, cell and medium isolates. cEPS, capsular EPS; FD, freeze‐dried; fEPS, free EPS; SD, spray‐dried; tEPS, total EPS. Subscript characters indicate pore sizes of microfiltration and ultrafiltration membranes used for the respective fEPS isolates

To obtain isolates containing EPS, bacterial cells and/or medium components, the fermented medium was diluted 1:2 with 9 g/L aqueous sodium chloride containing 0.2 g/L sodium azide to reduce medium viscosity and to prevent microbial growth. The subsequent isolation steps followed the scheme presented in Figure 1.

fEPS (free EPS) fractions (fEPS_0.1_5_, fEPS_0.45_5_ and fEPS_0.1_30_): Cells were removed by crossflow filtration at 40°C through 0.1 µm (polyethersulfone) or 0.45 µm (Hydrosart) membranes using Sartocon slice cassettes (Sartorius Stedim Biotech GmbH, Göttingen, Germany). The permeate was concentrated and purified (removal of mono‐ and disaccharides, amino acids and small peptides, salts and other minor components) by ultrafiltration and subsequent diafiltration through 5 kDa or 30 kDa polyethersulfone membranes. EPS precipitation was carried out by adding two volume units of cold acetone to the EPS containing retentate. After 24 h at 6°C, EPS were separated by centrifugation (19,000 g, 15 min, 4°C), resuspended in demineralised water and freeze‐dried (FD) (Alpha 1–2, Martin Christ Gefriertrocknungsanlagen, Osterode am Harz, Germany).

tEPS (total EPS): After initially releasing capsular EPS (cEPS) from the bacterial cells by heating the fermented medium to 90°C for 10 min and subsequent cooling to room temperature in an ice bath, the application of the procedure for fEPS (0.1 µm/5 kDa membranes) results in tEPS.

cEPS: Adding a very first removal of fEPS by crossflow microfiltration through 0.1 µm polyethersulfone membranes to the tEPS scheme results in the cEPS fraction.

Cells isolates: The cell containing microfiltration retentate was diafiltered and FD, resulting in a cell isolate with attached cEPS. To obtain cells without cEPS, the fermented medium was heated prior to microfiltration and then processed in the same way.

Medium isolates: The fermented medium was concentrated and dialyzed using 5 kDa polyethersulfone membranes and then FD. In addition, medium concentrates were prepared by spray drying (SD) using a B‐290 dryer equipped with a high‐performance cyclonic separator (BÜCHI Labortechnik AG, Flawil, Switzerland) at 140°C (inlet temperature), 960 mL/h concentrate flow (60% pump setting), a spray gas flow of 742 L/h and a gas flow of approx. 35 m^3^/h, resulting in a product outlet temperature between 58 and 62°C.

All isolates were weighed to calculate the yield [mg isolate/kg fermented medium], and stored in a desiccator until further use.

To obtain the exact EPS content of the fermented media, fEPS and tEPS isolation was performed in analytical (5 mL) scale as outlined by Mende et al. [29]. For fEPS isolation, the heating step was omitted.

### 2.3 Composition and macromolecular properties of EPS isolates

Moisture and ash were determined by thermogravimetry [30]. Total carbohydrate content, expressed as glucose equivalents [mg/L], was determined with the phenol sulphuric acid method [31]. Residual mono‐ and disaccharides were quantified by HPLC‐RI [30].

EPS molecular mass distribution was determined by size exclusion chromatography (AZURA Assistant ASM 2.1L) and RI detection (Smartline 2300, Knauer Wissenschaftliche Geräte GmbH, Germany) [28], and weight average m_M_ and number average molecular mass m_N_ [Da] was calculated.

To detect functional groups, all isolates were analysed by Fourier transform infrared spectroscopy (FT‐IR) with attenuated total reflection technology (Thermo Scientific FT‐IR Nicolet iS5 with iD5‐element, diamond crystal, Dreieich, Germany).

Dynamic viscosity η [mPa∙s] of aqueous EPS solutions at different concentrations c [g/L] was determined at 20°C using a LOVIS rolling ball viscometer (Anton Paar GmbH, Ostfildern, Germany). Using flow curves recorded with an AR‐G2 rheometer (TA Instruments GmbH, Eschborn, Germany) equipped with a concentric cylinder device (d_i_ = 28 mm, d_a_ = 30 mm, h = 42 mm), coil overlap concentrations c* and c** [g/L] were determined from double logarithmic plots of zero shear specific viscosity vs. EPS concentration [32]. The Huggins equation was used to calculate intrinsic viscosity [η] [mL/mg] of a solvent/polysaccharide pair as described previously [28].

Moisture sorption of EPS isolates was determined using a Q5000SA dynamic vapour sorption analyser (TA Instruments GmbH, Eschborn, Germany) [33]. Approx. 5 mg of sample were exposed to a relative humidity (r.h.) increase from 0 to 98% in 10% steps and a subsequent decrease to 0% [28]. The moisture load X [g H_2_O/g dry matter] at each r.h. was calculated from mass at equilibrium. Adsorption and desorption isotherms were generated from plots of X vs. r.h., and hysteresis areas are expressed in arbitrary units [AU].

All measurements were performed in duplicate.

### 2.4 Application of EPS isolates in model foods

The functionality of the EPS was determined in (I) chemically acidified milk gels, a model system that represents fermented products such as yoghurt, and in (II) emulsions, a non‐fermented, non‐dairy model system as potential future application for isolated EPS.

#### 2.4.1 Chemically acidified milk gels

Skim milk powder was dissolved in deionised water at 150 g/kg dry matter. After adding 0.3 g/kg sodium azide and storing overnight at 8°C for protein hydration, the reconstituted skim milk was heated at 90°C for 10 min and cooled to 30°C in an ice bath. Subsequently, an EPS, medium or cell isolate stock solution/suspension was added to achieve a final milk dry matter of 120 g/kg and isolate concentrations of 0–0.65 g/kg (EPS and medium isolates) or 0–10 g/kg (cell isolates).

Chemical acidification was induced by adding 300 mg glucono‐δ‐lactone to 10 mL reconstituted skim milk/EPS mixture at 30°C. Gelation of 360 µL aliquots was recorded with a MultiTEM:a thromboelastometer (SycoMed e.K., Lemgo, Germany) for approx. 150 min in triplicate [28], and pH was recorded simultaneously. The amplitude A_120_ [mm] of the gelation curve at 120 min was used as a measure for gel stiffness and normalized to A_120_ of reconstituted skim milk without EPS (blank).

#### 2.4.2 Model emulsions

Oil‐in‐water emulsions were prepared using the formulation of Håkansson et al. [34]. fEPS_0.1_5_, fEPS_0.45_5_, cEPS or tEPS were dissolved in demineralized water with 50 g/kg Tween^®^ 80 to achieve an EPS concentration of 8.1 g/kg. After addition of canola oil at a volume fraction of 52 %, the system was homogenized using an Ultra‐Turrax^®^ T25 with an S25N‐10G dispersing tool (IKA®‐Werke GmbH und CO. KG, Staufen, Germany) at 21500 rpm for 90 s. Emulsions without added EPS isolates served as reference.

Droplet size distributions were determined by laser diffraction spectroscopy using a HELOS/KR (λ = 633 nm, Sympatec GmbH, Clausthal‐Zellerfeld, Germany) with dispersing system CUVETTE 6 at an optical density of 10 – 25% in a measuring range of 0.1–35 µm. Sauter mean diameter d_3,2_ [µm] was obtained from the droplet size distributions calculated by the Mie theory using the refractive index of 1.47 (real part) and 0.01 (imaginary part) for canola oil [35].

Emulsion flow curves were recorded at 20°C with the AR‐G2 rheometer equipped with a parallel plate geometry (diameter = 40 mm, gap = 0.5 mm). Shear rate was stepwise increased from 0.001–100/s in logarithmic spacing (5 points/decade), and shear stress τ [Pa] at each shear rate was recorded for 10 s after 20 s equilibration. Apparent viscosity η_A_ [Pa⋅s] was calculated from τ at a shear rate of 100/s.

Emulsion stability was investigated with an analytical photo‐centrifuge LUMiSizer^®^ 610 (LUM GmbH, Berlin, Germany). 400 µL emulsion was transferred into rectangular polycarbonate cuvettes (2 × 8 mm^2^ base area), and transmission profiles were recorded at 1200 g and 25°C for 40 min. Creaming velocity v_C_ distribution was calculated with the SEPView^®^ software at positions of 124 mm, 126.5 mm, 128 mm (width = 1 mm) and the median v_C,50%_ [µm/s] was taken as stability indicator.

### 2.5 Statistical data evaluation

SAS^®^ University Edition 6p.2 (SAS^®^ Institute, Cary, NC, USA) was used for univariate ANOVA (*p* < 0.05) on sauter mean diameter, apparent viscosity at 100/s and median creaming velocity of model emulsions. Unless stated otherwise, data are expressed as arithmetic mean ± half deviation range (n = 2) or arithmetic mean ± standard deviation (n > 2).

## 3 RESULTS AND DISCUSSION

### 3.1 Composition of isolates

In the given fermentation medium, *S. thermophilus* DGCC7710 produced 892 mg/L fEPS and 1024 mg/L tEPS. The cEPS content refers to the difference tEPS – fEPS = 132 mg/L. These values served as reference for the evaluation of the isolation procedures in 1 L scale.

**FIGURE 2:**
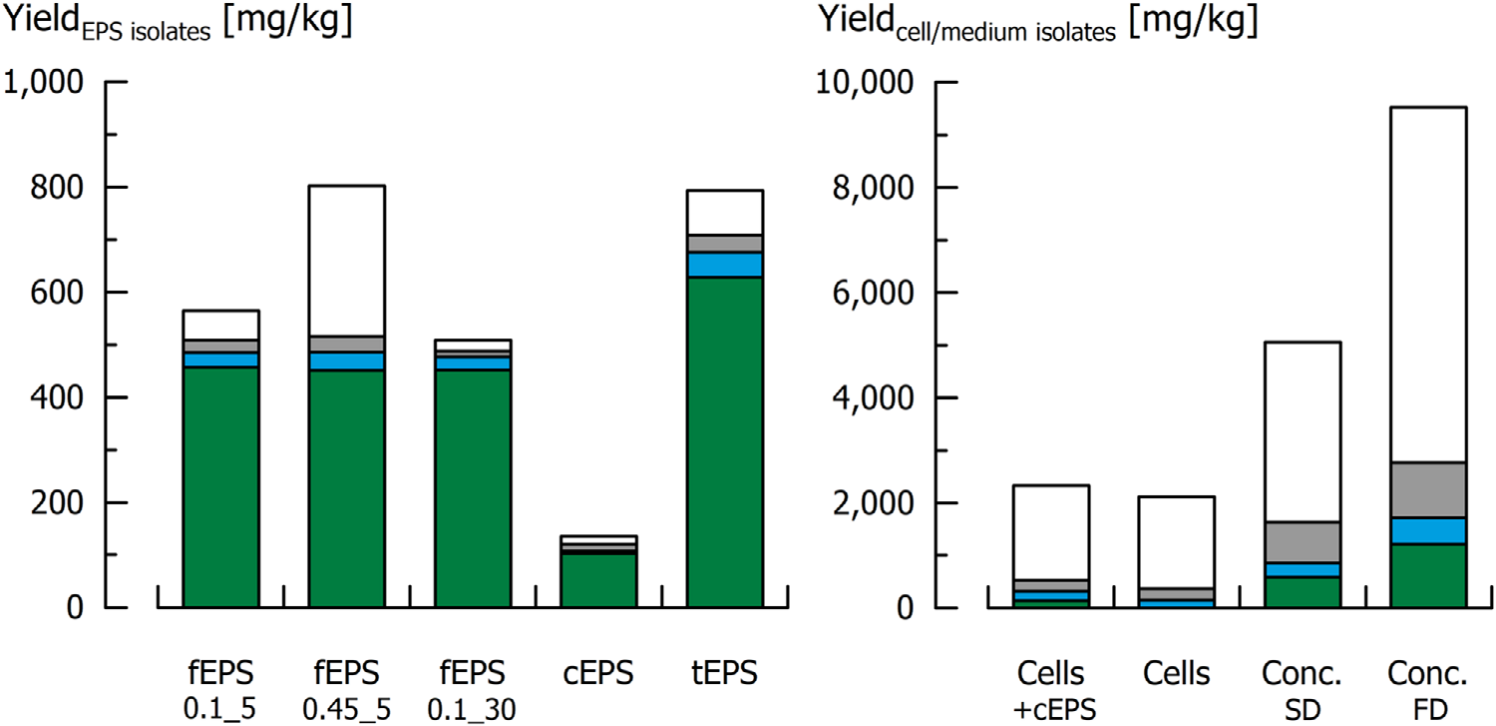
Yield [mg/kg fermented medium] and composition of different EPS, cell and medium isolates. Green: EPS, blue: water, grey: ash; white: not determined. Conc., medium concentrate; FD, freeze‐dried; SD, spray‐dried. For the different isolate abbreviations, please refer to Figure 1

The isolation of fEPS by microfiltration and ultrafiltration with membranes of different pore size (see also Figure 1) resulted in 564 (fEPS_0.1_5_), 802 (fEPS_0.45_5_) and 508 mg (fEPS_0.1_30_) isolate per kg fermented medium (Figure 2, left). Compositional analyses revealed that the absolute EPS amount was similar in all fEPS isolates (451 – 457 mg/kg), meaning that it was possible to isolate 51% of the free EPS with these procedures. For cEPS and tEPS, 78% (103 mg/kg) and 61% (628 mg/kg) of the EPS were isolated, respectively. The mono‐ and disaccharide content was below the detection limit in all EPS isolates, and the sum of moisture and ash was 7.1 – 13.2 g/100 g. Further impurities result from medium components that were co‐precipitated, e.g. peptides from tryptone. Thus, purities ( = relative EPS content of the isolates) of 80.9, 56.2 and 89.0% were obtained for fEPS_0.1_5_, fEPS_0.45_5_ and fEPS_0.1_30_, respectively, and of 75.8% for cEPS and 79.2% for tEPS. The use of microfiltration membranes with larger pore size (0.45 instead of 0.1 µm) accelerated the filtration process by approx. one third. This did not affect EPS yield (51%), but the purity of the isolates was, however, decreased. A 30 kDa ultrafiltration membrane (instead of 5 kDa) was found to be suitable to improve isolate purity from 80.9% (fEPS_0.1_5_) or 56.2% (fEPS_0.45_5_) to 89.0% (fEPS_0.1_30_).

As the use of highly purified hetero EPS is currently not cost‐efficient [13], partly purified, cell‐containing isolates were analysed. Due to the high cell mass, isolate yields of 2118 – 9518 mg/kg were obtained (Figure 2, right). To investigate the impact of cEPS bound to the bacterial cells, cell isolates with and without cEPS were examined. The purity (relative EPS amount) was low for cells with cEPS (6.2%), and consequently zero for cell isolates without cEPS. So called medium isolates were obtained by directly applying concentration, dialysis and drying of the entire fermented medium (Figure 1). Consequently, the medium isolates contain fEPS, which results in a purity of approx. 12% independent from the drying procedure (spray drying or freeze‐drying). Mono‐ and disaccharides were neither detected in cell nor in medium isolates, and the biomass present in the isolates caused the ash content to increase up to 15.5 g/100 g.

**FIGURE 3:**
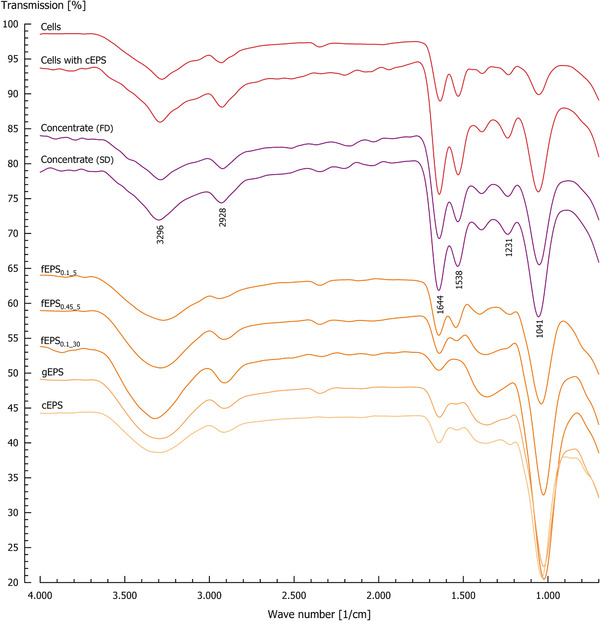
Infrared spectra of different EPS, cell and medium isolates. FD, freeze‐dried, SD, spray‐dried. Peak positions are exemplarily marked for the spray‐dried medium concentrate. For the different isolate abbreviations and colours, please refer to Figure 1

The composition of the isolates is also reflected by their FT‐IR spectra (Figure 3). The intense broad peak at approx. 3300/cm observed for all isolates corresponds to the ν(O–H) stretching vibration [36, 37]. The absorption band at 2928/cm was much more pronounced for cell‐containing isolates and can be attributed to the stretching of ν(C–H) from polysaccharides or fatty acids from cell membranes [38, 39]. Furthermore, the cell‐containing isolates showed pronounced peaks at 1644, 1538 and 1231/cm that were assigned to the amide I, II and III bonds and thus related to the protein content of the isolates [40, 41]. For EPS isolates, the amide I bond was less pronounced and smallest for fEPS_0.1_30_. This corresponded with the lowest impurity of the isolate (isolate purity: 89%). The pronounced peak at 1041/cm resulted from ν(C–O–C) stretching vibrations of glycosidic bonds, overlapped by ν(C–OH)/ δ(C–OH) stretching and bending vibrations [40, 42]. This typical peak for polysaccharides was more pronounced for isolates with higher purity (e.g. fEPS_0.1_30_).

### 3.2 Molecular mass distribution

The next step was to determine the molecular mass of the EPS present in the isolates. The EPS showed an m_M_ of approx. 2–3 × 10^6^ Da, a value typical for microbial heteropolysaccharides (Table 1) [11] and similar to m_M_ of the EPS determined after analytical isolation, indicating that no significant structural disruption occurred during pilot scale isolation. The weight average molecular mass m_M_ represents the fraction of larger molecules and was slightly higher for fEPS_0.45_5_ (3.29 × 10^6^ Da) than for fEPS_0.1_5_ (2.98 × 10^6^ Da). Additionally, fEPS_0.45_5_ are more polydisperse (polydispersity index Đ = 2.53) than fEPS_0.1_5_ (Đ = 2.06). This can be attributed to the different pore sizes of membranes used for cell separation. The 0.1 µm microfiltration membranes block more easily and may impede the transfer of larger EPS molecules into the permeate. Changing the ultrafiltration membrane (isolation of fEPS_0.1_30_) had only little effect on the molecular mass distribution: m_M_ was slightly lower, but m_N_ remained constant.

**TABLE 1:** Macromolecular properties of EPS isolates in aqueous solutions

	fEPS_0.1_5_	fEPS_0.45_5_	fEPS_0.1_30_	cEPS	tEPS
Molecular mass distribution					
m_M_ [10^6^ Da]	2.98 ± 0.12 ^a^	3.29 ± 0.10	2.57 ± 0.07	1.74 ± 0.09	2.61 ± 0.07
m_N_ [10^6^ Da]	1.45 ± 0.10	1.30 ± 0.06	1.46 ± 0.04	0.80 ± 0.02	1.29 ± 0.03
Đ = m_M_/m_N_ [−]	2.06^a^	2.53	1.76	2.17	2.02
Hydrodynamic radius R_h_ [nm]	61.7^a^	64.9	62.1	49.3	56.6
Critical overlap concentrations					
c* [g/L]	0.66	0.64	n.d.	n.d.	1.11
c** [g/L]	3.91	2.18	n.d.	n.d.	3.90

c*; c**, Critical overlap concentrations; Đ, Polydispersity index [‐]; m_M_, Weight average molecular mass [Da]; m_N_, Number average molecular mass [Da]; n.d., Not determined. For the different isolate abbreviations, please refer to Figure 1.

^a^ Published previously in Nachtigall et al. [28].

The molecular mass of cEPS was lower (m_M_ = 1.74 × 10^6^ Da) than that of fEPS, but in the same order of magnitude. In studies with *S. thermophilus* ST‐143, Mende et al. [20] found significantly lower m_M_ for two cEPS fractions (approx. 10^5^ and 10^3^ Da) compared with fEPS (m_M_ approx. 10^6^ Da). It remains still unclear whether cEPS have a lower molecular mass in general, or whether a molecular breakdown is likely during isolation, especially during heat treatment. The thermal treatment was applied as short as possible (10 min at 90°C) and cannot be omitted, when cEPS should be removed from the cells. Alternative treatments for cEPS detachment such as ultrasonication are not appropriate as they also induce the breakdown of covalent bonds [43, 44, 45]. As a consequence, tEPS as a mixture of fEPS and cEPS showed a molecular mass distribution between both individual EPS fractions (Table 1).

To determine the molecular mass of EPS in medium isolates, they were suspended in deionised water, followed by centrifugation for cell removal and dialysis against deionised water. We found that the molecular mass distribution was neither altered by spray drying nor by freeze drying, which is in line with a study on EPS from *Lactobacillus helveticus* where even higher inlet (160°C) and outlet (80°C) temperatures were applied during spray drying compared to our study (140°C and 58–62°C, respectively) [37]. For plant polysaccharides, however, a significant decrease of the molecular mass was observed already at lower temperatures by using spray drying with a rotary atomizer (inlet temperature 150°C and outlet temperature 78°C) or drying in an oven (12 h at 75°C) [46, 47]. For hot air drying performed at 50°C for 5 h, this was attributed to the rapid removal of bound water causing a breakdown of the polysaccharide structure [48].

### 3.3 Rheological properties of aqueous EPS solutions

Molecular mass, among others, significantly affects the behaviour of macromolecules in solution. In a previous study, [η] = 0.543 mL/mg was calculated for fEPS_0.1_5_ [28]. This value was confirmed for large scale isolation, and other isolates showed similar [η] of 0.526 mL/mg (fEPS_0.45_5_) and 0.588 mL/mg (fEPS_0.1_30_). For cEPS and tEPS isolates, [η] was lower (0.433 and 0.438 mL/mg, respectively). As the molecular mass of cEPS is in the same order of magnitude as fEPS and we assume that the chemical structure for fEPS and cEPS is identical because of a similar synthesis pathway [49], the difference in [η] is likely to be caused by the heat treatment (90°C for 10 min) during isolation. Similar results are evident from literature: for algae polysaccharide solutions from *Porphyridium* ssp. the drying process affected, among others, intrinsic viscosity, but had an only negligible effect on molecular mass [50]. Similarly, for κ‐carrageenan and agarose, [η] was lower after a heat treatment [51]. Wang et al. [52] concluded that small aggregates of different uncharged polysaccharides with defined molecular mass distributions cause lower [η] than larger aggregates. It can be concluded that protein or peptide remnants from the fermentation medium do not affect [η] of EPS in aqueous solution, but that a heat treatment leads to reduced EPS/solvent interactions.

With m_M_, [η] and the Avogadro constant N_A_, the hydrodynamic radius R_h_ can be calculated as follows [53]. For the three fEPS isolates from different isolation procedures, R_h_ differed only slightly (61.7 – 64.9 nm) as a consequence of nearly unchanged m_M_ and [η] (Table 1). Under the assumption of perfect spheres, the molecules occupied a volume of 9.8 × 10^5^ – 11.5 × 10^5^ nm^3^. With smaller R_h_, this volume was reduced for cEPS and tEPS to 5.0 × 10^5^ and 7.6 × 10^5^ nm^3^, respectively.

For selected EPS isolates, coil overlap concentrations c* and c** which split the concentration range into fully diluted (individual molecules), semi‐diluted (overlapping molecules) and concentrated (entangled molecules) solution [32, 54], were determined. For fEPS_0.1_5_, the semi‐diluted region was found at 0.66 < c < 3.91 g/L (Table 1). With decreasing EPS content, more interactions between EPS molecules and isolate impurities occurred, and c** decreased for fEPS_0.45_5_ to 2.18 g/L. For tEPS with an EPS content similar to that of fEPS_0.1_5_ (approx. 80%) c** was not altered, indicating that the thermal treatment did not affect the entanglement of the molecules. The present results fit in the range of values for other EPS from lactic acid bacteria [20, 30, 55, 56].

### 3.4 Moisture sorption

**FIGURE 4:**
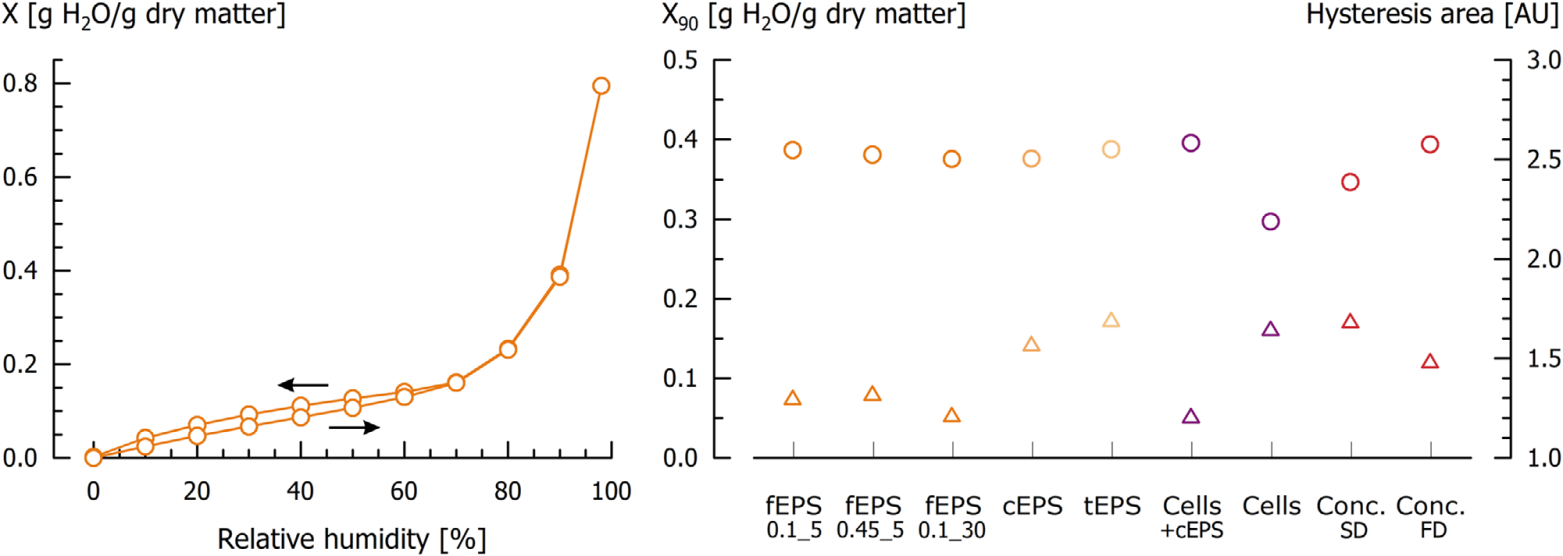
Moisture adsorption and desorption isotherms of fEPS_0.1_5_ from *S. thermophilus* DGCC7710 (left chart: moisture load X [g H_2_O/g dry matter]), and moisture load at 90% relative humidity X_90_ (circles) and hysteresis area [AU] of different isolates at a_w_ = 0.90 (triangles) (right chart). For the different isolate abbreviations and colours, please refer to Figure 1

One of the most important technofunctional properties of polymeric carbohydrates is the capacity to immobilise water. The sorption isotherms of all isolates showed a sigmoid curve progression, typical for foods and related samples (Figure 4, left). At low r.h., only small amounts of moisture adsorbed. At r.h. > 70%, moisture adsorption was increased because of the presence of salts or (poly)saccharides [57, 58].

The comparison of the moisture loads at r.h. = 90% shows that EPS isolates (fEPS_0.1_5_, fEPS_0.45_5_, fEPS_0.1_30_, cEPS, tEPS) have a nearly similar X_90_, ranging from 0.376 – 0.392 g/g (Figure 4, right). This suggests that the absolute polysaccharide content of the isolates is not decisive for the amount of adsorbed moisture. Cells covered with cEPS showed a similar X_90_, and the removal of cEPS from the cells led to a pronounced decrease (X_90_ = 0.297 g/g). We assume that the chemical structure of fEPS and cEPS in this study is similar, because altered X_90_ values were found for EPS from different *S. thermophilus* strains [20, 30]. This indicates that cEPS play an important role for the cell surface properties in fermented dairy products through enhancing the water binding capacity compared to cEPS negative strains. The FD medium concentrate also showed a similar X_90_ whereas, for the spray‐dried (SD) concentrates, X_90_ was 0.347 g/g, probably because cEPS were detached from the cell wall during spray‐drying. This was confirmed by light microscopy, so that a more hydrophobic cell surface is exposed. As the detached cEPS are still present in the isolate, the X_90_ was higher than of cells without EPS, but lower compared to the EPS isolates.

Furthermore, the isolates differed in the hysteresis area enclosed between adsorption and desorption isotherms (Figure 4, right). Isolates subjected to a thermal impact during isolation (cEPS, tEPS, cells without cEPS, SD medium concentrate) showed higher hysteresis areas (1.58 – 1.70 AU) than isolates without thermal treatment (all fEPS isolates, cells with cEPS, FD medium concentrate; 1.21 – 1.49 AU). The appearance of hystereses is generally related to re‐arrangements in the system and impeded moisture desorption. Therefore, more energy is necessary to remove moisture from the matrix, for example because hydrophilic groups are not exposed to the outer surface of the molecules [59, 60]. After desorption, moisture load was not exactly zero again, suggesting that some of the bound moisture could not be desorbed in the time span of the experiment [57].

### 3.5 How different isolates affect physical properties of chemically acidified milk

**FIGURE 5:**
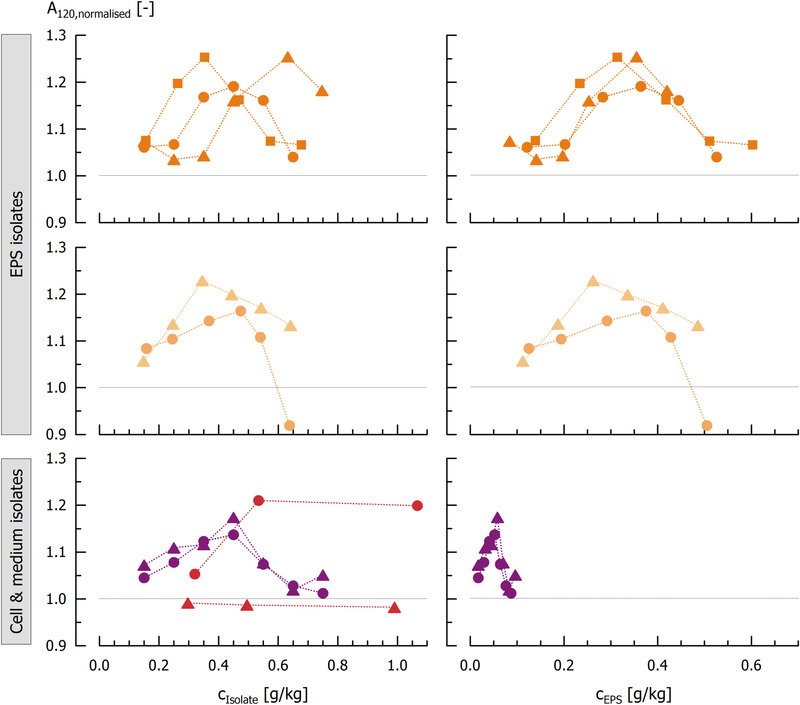
Normalised gel stiffness A_120,normalised_ [‐] of acidified milk gels supplemented with EPS, cell and medium isolates prior to acidifaction as affected by the amount of added isolate (left) and as affected by EPS concentration, calculated using the relative EPS content of the respective isolate. Dark orange: fEPS_0.1_5_ (circles); fEPS_0.45_5_ (triangles) and fEPS_0.1_30_ (squares); light orange: tEPS (circles) and cEPS (triangles); violet: freeze‐dried concentrate (circles) and spray‐dried concentrate (triangles); red: cells with cEPS (circles) and cells without cEPS (triangles). For the different isolate abbreviations, please refer to Figure1

The addition of EPS prior to acidification generally increased the stiffness of acid milk gels (A_120,normalised_), whereas cell isolates without cEPS did not affect A_120,normalised_ at all (Figure 5). For the EPS isolates, gel stiffness A_120,normalised_ increased with isolate concentration until a critical concentration (0.35 – 0.65 g/kg for the different isolates). Above this concentration, A_120,normalised_ decreased again. This may be explained by the different mechanism of EPS embedment in the gel network, compared to *in situ* formation: in our model system, EPS concentration is constant during the entire gelation and, thus, may impede the formation of a solid three‐dimensional protein network at high concentrations. Girard and Schaffer‐Lequart [61] observed that ropy EPS impeded structure recovery of sheared milk gels because they disrupted the interactions between protein particles. In our study, A_120,normalised_ did not decrease at higher concentrations for cell isolates, indicating that the cells are embedded more evenly in the protein network without affecting gel stiffness.

The highest A_120,normalised_ were observed for fEPS_0.1_30_ and fEPS_0.45_5_ (A_120,normalised_ = 1.25). As the isolates had different purities, data presented in Figure 5 (left) were redrawn by considering the absolute EPS amount added to reconstituted skim milk prior to acidification (Figure 5, right). It is evident that all gels showed an A_120,normalised_ maximum at approx. 0.4 g pure EPS/kg. Thus, the effectiveness of the isolates in milk gels depends on the absolute EPS content, whereas non‐EPS substances do not impede the functionality of the isolate. With regard to the amount of added isolate needed for the highest possible A_120,normalised_, isolates with high purity are preferred such as fEPS_0.1_30_ in our study.

Despite their low EPS amount (maximum at 0.06 g EPS/kg), medium isolates increased A_120,normalised_ up to 1.14 (FD) and 1.17 (SD). We assume that bacterial cells and cEPS still present in the medium isolate contributed to the increase in gel stiffness. This was confirmed after adding cell isolates to reconstituted skim milk: cells with attached cEPS increased A_120,normalised_ in contrast to cells without cEPS, indicating that cell bound cEPS also contribute to the technofunctionality of milk gels. This is also discussed in literature, as cells can interact with EPS or act as fillers in the pores of the protein network [21, 62].

### 3.6 Application of isolates in model O/W emulsions

**FIGURE 6:**
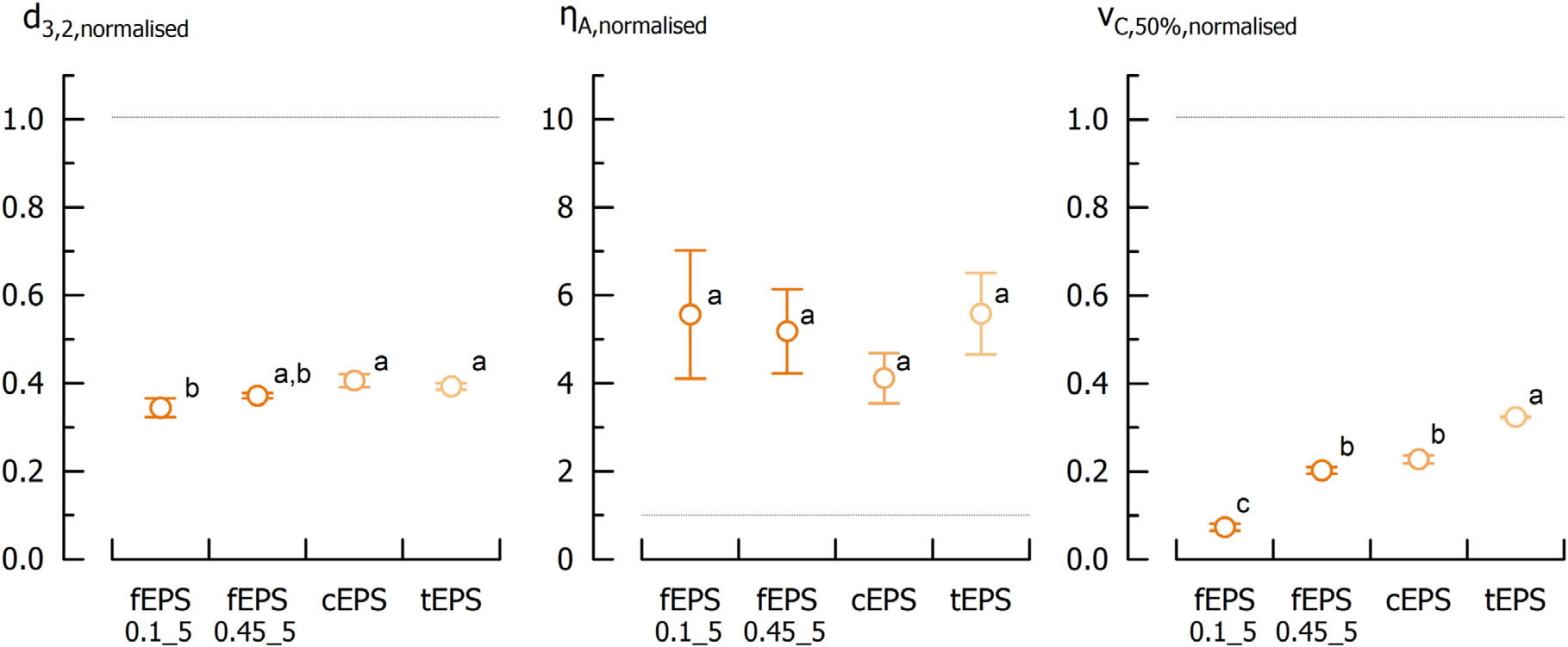
Normalised Sauter mean diameter d_3,2,normalised_, apparent viscosity at 100/s η_A,normalised_ and median of creaming velocity distribution v_C,50%,normamlised_ of model O/W emulsions with EPS isolates (EPS concentration = 8.1 g/kg). The dotted line indicates the respective property of emulsions without isolates (for absolute values, see section 3.6). Superscripts with different letters within the same graph are significantly different (*p* < 0.05). For the different isolate abbreviations and colours, please refer to Figure 1

To illustrate the effect of selected EPS isolates (fEPS_0.1_5_, fEPS_0.45_5_, cEPS, and tEPS) on the stability of O/W emulsions, an EPS concentration of 8.1 g/kg in the aqueous phase (above c**) was chosen; this corresponds to an amount of c_Isolate_ = 10 g/kg of fEPS isolates. A c_Isolate_ of 10 g/kg was also used for emulsions with uncharged EPS from *Bifidobacterium longum* ssp. *infantis* by Prasanna et al. [25]. Sauter mean diameter, apparent viscosity at 100/s and median of creaming velocity distribution of model emulsions with EPS isolates were normalised to the respective data of emulsions without isolates (d_3,2_ = 4.8 ± 0.1 µm, η_A_ = 0.022 ± 0.002 mPa⋅s, v_C,50%_ = 37 ± 1 µm/s). The use of isolates in the emulsions resulted in a significantly lower Sauter mean diameter and creaming velocity and a higher apparent viscosity, which implies sufficient emulsification properties (Figure 6). Emulsions with fEPS_0.1_5_ revealed the lowest d_3,2_, and a high η_A_ indicates enhanced physical emulsion stability. This was confirmed by sedimentation analysis where v_C,50%_ was significantly lower than for the other isolates. The lower emulsion stability with cEPS and tEPS (high v_C,50%_ and d_3,2_) compared to fEPS_0.1_5_ can be explained by the thermal impact during isolation that resulted in a lower hydrodynamic radius, and therefore in a lower functionality in the emulsions. This is in agreement with results on gel stiffness of acid milk gels, where the lower functionality of tEPS resulted in a lower A_120,normalised_ than for cEPS. fEPS_0.45_5_ revealed a faster phase separation (higher v_C,50%_) than fEPS_0.1_5_ which might be attributed to its lower purity (56 *vs*. 81%, respectively). The lower purity of fEPS_0.45_5_ was attributed to the presence of co‐precipitated impurities, e.g. partly digested proteins, as ash and water content were similar for these isolates. Generally, proteins can interact with the polysaccharides and/or droplet surface, and therefore may cause instabilities [63]. Because of the high purity and functionality of fEPS_0.1_5_, this isolate is better capable to prevent creaming of emulsions than cEPS, tEPS and fEPS_0.45_5_. That uncharged EPS with a purity above 80% contributed to smaller droplets similar to guar gum and xanthan was demonstrated recently for EPS from *Bifidobacterium longum* ssp. *infantis* [25].

## 4 CONCLUDING REMARKS

Variations of the isolation procedure are responsible for significant changes in macromolecular and technofunctional properties of the respective EPS isolates from *S. thermophilus* DGCC7710: (1) Microfiltration membranes with larger pores (0.45 instead of 0.1 µm) reduce isolation time, but result in a higher amount of non‐EPS substances in the isolates (EPS content was lowered from 81 to 56%) and a shift of the coil overlap to lower EPS concentration. The absolute EPS yield remained constant at 51% for all fEPS isolates. (2) Ultrafiltration membranes with larger pores (30 instead of 5 kDa) increase isolate purity (89%). (3) An additional heating step detaches cEPS from the cells and therefore increase EPS yield, but decrease intrinsic viscosity and molecular mass of EPS in the isolates. (4) The production of cell containing isolates and medium concentrates shortens isolation time considerably and results in large amounts of isolates containing approx. 88% of non‐EPS substances. The molecular mass of the EPS was not affected by the applied spray drying or freeze‐drying procedure. A higher moisture load of cells with cEPS (similar to EPS isolates) compared to cEPS‐free cells was observed. This emphasises the importance of cEPS for the water binding capacity in fermented products.

When added to reconstituted skim milk prior to acidification, all EPS isolates provoked a concentration dependent increase in gel stiffness (+25% at maximum) until a critical concentration of approx. 0.4 g EPS/kg. The content of non‐EPS substances in these isolates did not affect gel stiffness. Furthermore, the results indicate the high potential of fEPS in isolates with a purity > 80% for stabilising emulsions because of the ability to contribute to small droplets and low creaming velocity. Medium isolates with cells covered by cEPS showed similar effects in milk gels although the absolute EPS concentrations were very low (approx. 0.1 g/kg). In combination with their simple isolation protocols, this makes them highly suitable for the use in non‐fermented products.

## CONFLICTS OF INTEREST

The authors have declared no conflicts of interest.

**Nomenclature**

**Table:** 

[η]	[mL/mg]	Intrinsic viscosity
A_120_	[mm]	Gel stiffness of acidified milk
c*; c**	[g/L]	Critical overlap concentrations
Đ	[‐]	Polydispersity index
d_3,2_	[µm]	Sauter mean diameter
m_M_	[Da]	Weight average molecular mass
m_N_	[Da]	Number average molecular mass
R_h_	[nm]	Hydrodynamic radius
r.h.	[%]	Relative humidity
v_C,50%_	[µm/s]	Median of creaming velocity distribution
X	[g H_2_O/g dry matter]	Moisture load
η	[mPa·s]	Dynamic viscosity
η_A_	[Pa·s]	Apparent viscosity at a shear rate of 100/s
τ	[Pa]	Shear stress

## Data Availability

Research data are not shared.

## References

[1] Ngouémazong, E. D. , Christiaens, S. , Shpigelman, A. , Van Loey, A. , et al., The emulsifying and emulsion‐stabilizing properties of pectin: A review. Compr. Rev. Food Sci. Food Saf. 2015, 14, 705–718.

[2] Zia, K. M. , Tabasum, S. , Nasif, M. , Sultan, N. , et al., A review on synthesis, properties and applications of natural polymer based carrageenan blends and composites. Int. J. Biol. Macromol. 2017, 96, 282–301. 27914965 10.1016/j.ijbiomac.2016.11.095

[3] Singh, R. S. , Saini, G. K. , Kennedy, J. F. , Pullulan: Microbial sources, production and applications. Carbohydr. Polym. 2008, 73, 515–531. 26048217 10.1016/j.carbpol.2008.01.003

[4] McKim, J. M. , Food additive carrageenan: Part I: A critical review of carrageenan *in vitro* studies, potential pitfalls, and implications for human health and safety. Crit. Rev. Toxicol. 2014, 44, 211–243. 24456237 10.3109/10408444.2013.861797

[5] Weiner, M. L. , Food additive carrageenan: Part II: A critical review of carrageenan *in vivo* safety studies. Crit. Rev. Toxicol. 2014, 44, 244–269. 24467586 10.3109/10408444.2013.861798

[6] European Commission, E. Commission: 2001/122/EC. Commission decision of 30 January 2001 on authorising the placing on the market of a dextran preparation produced by *Leuconostoc mesenteroides* as a novel food ingredient in bakery products under Regulation (EC) No 258/97 of the European Parliament and of the Council, 2001.

[7] European Commission, Regulation (EC) No 1333/2008 of the European Parliament and of the Council of 16 December 2008 on food additives, 2008.

[8] Zannini, E. , Waters, D. M. , Coffey, A. , Arendt, E. K. , Production, properties, and industrial food application of lactic acid bacteria‐derived exopolysaccharides. Appl. Microbiol. Biotechnol. 2016, 100, 1121–1135. 26621802 10.1007/s00253-015-7172-2

[9] EFSA Panel on Biological Hazards (BIOHAZ) , Koutsoumanis, K. , Allende, A. , Alvarez‐Ordóñez, A. , et al., Update of the list of QPS‐recommended biological agents intentionally added to food or feed as notified to EFSA 11: Suitability of taxonomic units notified to EFSA until September 2019. EFSA J. 2020, 18, 5965. 10.2903/j.efsa.2020.5965 PMC7448003 32874211

[10] De Vuyst, L. , Vanderveken, F. , Van de Ven, S. , Degeest, B. , Production by and isolation of exopolysaccharides from *Streptococcus thermophilus* grown in a milk medium and evidence for their growth‐associated biosynthesis. J. Appl. Microbiol. 1998, 84, 1059–1068. 9717291 10.1046/j.1365-2672.1998.00445.x

[11] Lynch, K. M. , Zannini, E. , Coffey, A. , Arendt, E. K. , Lactic acid bacteria exopolysaccharides in foods and beverages: Isolation, properties, characterization, and health benefits. Annu. Rev. Food Sci. Technol. 2018, 9, 155–176. 29580141 10.1146/annurev-food-030117-012537

[12] Leroy, F. , De Vuyst, L. , Advances in production and simplified methods for recovery and quantification of exopolysaccharides for applications in food and health. J. Dairy Sci. 2016, 99, 3229–3238. 26874424 10.3168/jds.2015-9936

[13] Mende, S. , Rohm, H. , Jaros, D. , Influence of exopolysaccharides on the structure, texture, stability and sensory properties of yoghurt and related products. Int. Dairy J. 2016, 52, 57–71.

[14] Pintado, A. I. E. , Ferreira, J. A. , Pintado, M. M. E. , Gomes, A. M. P. , et al., Efficiency of purification methods on the recovery of exopolysaccharides from fermentation media. Carbohydr. Polym. 2020, 231, 115703. 31888825 10.1016/j.carbpol.2019.115703

[15] Rimada, P. S. , Abraham, A. G. , Comparative study of different methodologies to determine the exopolysaccharide produced by kefir grains in milk and whey. Lait 2003, 83, 79–87.

[16] Oleksy‐Sobczak, M. , Klewicka, E. , Piekarska‐Radzik, L. , Exopolysaccharides production by *Lactobacillus rhamnosus* strains – Optimization of synthesis and extraction conditions. LWT ‐ Food Sci. Techn. 2020, 122, 109055.

[17] Goh, K. K. T. , Haisman, D. R. , Singh, H. , Development of an improved procedure for isolation and purification of exopolysaccharides produced by *Lactobacillus delbrueckii* subsp. *bulgaricus* NCFB 2483. Appl. Microbiol. Biotechnol. 2005, 67, 202–208. 15480631 10.1007/s00253-004-1739-7

[18] Notararigo, S. , Nácher‐Vázquez, M. , Ibarburu, I. , Werning, M. L. , et al., Comparative analysis of production and purification of homo‐ and hetero‐polysaccharides produced by lactic acid bacteria. Carbohydr. Polym. 2013, 93, 57–64. 23465901 10.1016/j.carbpol.2012.05.016

[19] Mende, S. , Peter, M. , Bartels, K. , Rohm, H. , et al., Addition of purified exopolysaccharide isolates from *S. thermophilus* to milk and their impact on the rheology of acid gels. Food Hydrocoll. 2013, 32, 178–185.

[20] Mende, S. , Dong, T. , Rathemacher, A. , Rohm, H. , et al., Physicochemical characterisation of the exopolysaccharides of *Streptococcus thermophilus* ST‐143. Int. J. Food Sci. Technol. 2014, 49, 1254–1263.

[21] Girard, M. , Schaffer‐Lequart, C. , Gelation of skim milk containing anionic exopolysaccharides and recovery of texture after shearing. Food Hydrocolloids 2007, 21, 1031–1040.

[22] Petry, S. , Furlan, S. , Waghorne, E. , Saulnier, L. , et al., Comparison of the thickening properties of four *Lactobacillus delbrueckii* subsp. *bulgaricus* strains and physicochemical characterization of their exopolysaccharides. FEMS Microbiol. Lett. 2003, 221, 285–291. 12725940 10.1016/S0378-1097(03)00214-3

[23] Ruas‐Madiedo, P. , Tuinier, R. , Kanning, M. , Zoon, P. , Role of exopolysaccharides produced by *Lactococcus lactis* subsp. *cremoris* on the viscosity of fermented milks. Int. Dairy J. 2002, 12, 689–695.

[24] Kielak, A. M. , Castellane, T. C. L. , Campanharo, J. C. , Colnago, L. A. , et al., Characterization of novel *Acidobacteria* exopolysaccharides with potential industrial and ecological applications. Sci. Rep. 2017, 7, 41193. 28117455 10.1038/srep41193 PMC5259719

[25] Prasanna, P. H. P. , Bell, A. , Grandison, A. S. , Charalampopoulos, D. , Emulsifying, rheological and physicochemical properties of exopolysaccharide produced by *Bifidobacterium longum* subsp. *infantis* CCUG 52486 and *Bifidobacterium infantis* NCIMB 702205. Carbohydr. Polym. 2012, 90, 533–540. 24751074 10.1016/j.carbpol.2012.05.075

[26] Laneuville, S. I. , Turgeon, S. L. , Microstructure and stability of skim milk acid gels containing an anionic bacterial exopolysaccharide and commercial polysaccharides. Int. Dairy J. 2014, 37, 5–15.

[27] Yang, T. , Wu, K. , Wang, F. , Liang, X. , et al., Effect of exopolysaccharides from lactic acid bacteria on the texture and microstructure of buffalo yoghurt. Int. Dairy J. 2014, 34, 252–256.

[28] Nachtigall, C. , Berger, C. , Kovanović, T. , Wefers, D. , et al., Shear induced molecular changes of exopolysaccharides from lactic acid bacteria. Food Hydrocoll. 2019, 97, 105181.

[29] Mende, S. , Mentner, C. , Thomas, S. , Rohm, H. , et al., Exopolysaccharide production by three different strains of *Streptococcus thermophilus* and its effect on physical properties of acidified milk. Eng. Life Sci. 2012, 12, 466–474.

[30] Nachtigall, C. , Surber, G. , Herbi, F. , Wefers, D. , et al., Production and molecular structure of heteropolysaccharides from two lactic acid bacteria. Carbohydr. Polym. 2020, 236, 116019. 32172839 10.1016/j.carbpol.2020.116019

[31] Dubois, M. , Gilles, K. A. , Hamilton, J. K. , Rebers, P. A. , et al., Colorimetric method for determination of sugars and related substances. Anal. Chem. 1956, 28, 350–356.

[32] Morris, E. R. , Cutler, A. N. , Ross‐Murphy, S. B. , Rees, D. A. , et al., Concentration and shear rate dependence of viscosity in random coil polysaccharide solutions. Carbohydr. Polym. 1981, 1, 5–21.

[33] Passauer, L. , Struch, M. , Schuldt, S. , Appelt, J. , et al., Dynamic moisture sorption characteristics of xerogels from water‐swellable oligo(oxyethylene) lignin derivatives. ACS Appl. Mater. Interfaces 2012, 4, 5852–5862. 23075458 10.1021/am3015179

[34] Håkansson, A. , Chaudhry, Z. , Innings, F. , Model emulsions to study the mechanism of industrial mayonnaise emulsification. Food Bioprod. Process. 2016, 98, 189–195.

[35] Kenmogne‐Domguia, H. B. , Meynier, A. , Viau, M. , Llamas, G. , et al., Gastric conditions control both the evolution of the organization of protein‐stabilized emulsions and the kinetic of lipolysis during in vitro digestion. Food Funct. 2012, 3, 1302. 22918290 10.1039/c2fo30031a

[36] Du, B. , Zeng, H. , Yang, Y. , Bian, Z. , et al., Anti‐inflammatory activity of polysaccharide from *Schizophyllum commune* as affected by ultrasonication. Int. J. Biol. Macromol. 2016, 91, 100–105. 27189700 10.1016/j.ijbiomac.2016.05.052

[37] Xiao, L. , Li, Y. , Tian, J. , Zhou, J. , et al., Influences of drying methods on the structural, physicochemical and antioxidant properties of exopolysaccharide from *Lactobacillus helveticus* MB2‐1. Int. J. Biol. Macromol. 2020, 157, 220–231. 32344080 10.1016/j.ijbiomac.2020.04.196

[38] Benhouna, I. S. , Heumann, A. , Rieu, A. , Guzzo, J. , et al., Exopolysaccharide produced by *Weissella confusa*: Chemical characterisation, rheology and bioactivity. Int. Dairy J. 2019, 90, 88–94.

[39] Deepika, G. , Green, R. J. , Frazier, R. A. , Charalampopoulos, D. , Effect of growth time on the surface and adhesion properties of *Lactobacillus rhamnosus* GG. J. Appl. Microbiol. 2009, 107, 1230–1240. 19486400 10.1111/j.1365-2672.2009.04306.x

[40] Ren, W. , Xia, Y. , Wang, G. , Zhang, H. , et al., Bioactive exopolysaccharides from a *S. thermophilus* strain: Screening, purification and characterization. Int. J. Biol. Macromol. 2016, 86, 402–407. 26820354 10.1016/j.ijbiomac.2016.01.085

[41] Zhao, D. , Shah, N. P. , Effect of tea extract on lactic acid bacterial growth, their cell surface characteristics and isoflavone bioconversion during soymilk fermentation. Food Res. Int. 2014, 62, 877–885.

[42] Rosca, I. , Petrovici, A. R. , Peptanariu, D. , Nicolescu, A. , et al., Biosynthesis of dextran by *Weissella confusa* and its In vitro functional characteristics. Int. J. Biol. Macromol. 2018, 107, 1765–1772. 29030182 10.1016/j.ijbiomac.2017.10.048

[43] Chen, X. , Siu, K. ‐ C. , Cheung, Y. ‐ C. , Wu, J. ‐ Y. , Structure and properties of a (1→3)‐β‐d‐glucan from ultrasound‐degraded exopolysaccharides of a medicinal fungus. Carbohydr. Polym. 2014, 106, 270–275. 24721078 10.1016/j.carbpol.2014.02.040

[44] Li, J. , Li, B. , Geng, P. , Song, A. ‐ X. , et al., Ultrasonic degradation kinetics and rheological profiles of a food polysaccharide (konjac glucomannan) in water. Food Hydrocoll. 2017, 70, 14–19.

[45] Wang, Z. ‐ M. , Cheung, Y. ‐ C. , Leung, P. ‐ H. , Wu, J. ‐ Y. , Ultrasonic treatment for improved solution properties of a high‐molecular weight exopolysaccharide produced by a medicinal fungus. Bioresour. Technol. 2010, 101, 5517–5522. 20171885 10.1016/j.biortech.2010.01.134

[46] Medina‐Torres, L. , Calderas, F. , Minjares, R. , Femenia, A. , et al., Structure preservation of Aloe vera (*barbadensis Miller*) mucilage in a spray drying process. LWT ‐ Food Sci. Techn. 2016, 66, 93–100.

[47] Yuan, Q. , He, Y. , Xiang, P. ‐ Y. , Huang, Y. ‐ J. , et al., Influences of different drying methods on the structural characteristics and multiple bioactivities of polysaccharides from okra (*Abelmoschus esculentus*). Int. J. Biol. Macromol. 2020, 147, 1053–1063. 31756490 10.1016/j.ijbiomac.2019.10.073

[48] Wang, Y. , Li, X. , Zhao, P. , Qu, Z. , et al., Physicochemical characterizations of polysaccharides from Angelica Sinensis Radix under different drying methods for various applications. International Journal of Biological Macromolecules 2019, 121, 381–389. 30315881 10.1016/j.ijbiomac.2018.10.035

[49] Schmid, J. , Recent insights in microbial exopolysaccharide biosynthesis and engineering strategies. Curr. Opin. Biotechnol. 2018, 53, 130–136. 29367163 10.1016/j.copbio.2018.01.005

[50] Ginzberg, A. , Korin, E. , Arad, S. (Malis), Effect of drying on the biological activities of a red microalgal polysaccharide. Biotechnol. Bioeng. 2008, 99, 411–420. 17625787 10.1002/bit.21573

[51] Lai, V. M. ‐ F. , Lii, C. , Hung, W. ‐ L. , Lu, T. ‐ J. , Kinetic compensation effect in depolymerisation of food polysaccharides. Food Chem. 2000, 68, 319–325.

[52] Wang, Q. , Wood, P. J. , Cui, W. , Ross‐Murphy, S. B. , The effect of autoclaving on the dispersibility and stability of three neutral polysaccharides in dilute aqueous solutions. Carbohydr. Polym. 2001, 45, 355–362.

[53] Antoniou, E. , Themistou, E. , Sarkar, B. , Tsianou, M. , et al., Structure and dynamics of dextran in binary mixtures of a good and a bad solvent. Colloid. Polym. Sci. 2010, 288, 1301–1312.

[54] Robinson, G. , Ross‐Murphy, S. B. , Morris, E. R. , Viscosity‐molecular weight relationships, intrinsic chain flexibility, and dynamic solution properties of guar galactomannan. Carbohydr. Res. 1982, 107, 17–32.

[55] Goh, K. K. T. , Hemar, Y. , Singh, H. , Viscometric and static light scattering studies on an exopolysaccharide produced by *Lactobacillus delbrueckii* subspecies *bulgaricus* NCFB 2483. Biopolymers 2005, 77, 98–106. 15625727 10.1002/bip.20192

[56] Gorret, N. , Renard, C. M. G. C. , Famelart, M. H. , Maubois, J. L. , et al., Rheological characterization of the EPS produced by *P. acidi‐propionici* on milk microfiltrate. Carbohydr. Polym. 2003, 51, 149–158.

[57] Al‐Muhtaseb, A. H. , McMinn, W. A. M. , Magee, T. R. A. , Moisture sorption isotherm characteristics of food products: A review. Food Bioprod. Process. 2002, 80, 118–128.

[58] Mathlouthi, M. , Rogé, B. , Water vapour sorption isotherms and the caking of food powders. Food Chem. 2003, 82, 61–71.

[59] Andrade, P. R.D. , Lemus, M. R. , Pérez, C. C.E. , Models of sorption isotherms for food: Uses and limitations. Vitae 2011, 18, 325–334.

[60] Caurie, M. , Hysteresis phenomenon in foods. Int. J. Food Sci. Technol. 2007, 42, 45–49.

[61] Girard, M. , Schaffer‐Lequart, C. , Gelation and resistance to shearing of fermented milk: Role of exopolysaccharides. Int. Dairy J. 2007, 17, 666–673.

[62] De Vuyst, L. , Zamfir, M. , Mozzi, F. , Adriany, T. , et al., Exopolysaccharide‐producing *Streptococcus thermophilus* strains as functional starter cultures in the production of fermented milks. Int. Dairy J. 2003, 13, 707–717.

[63] Dickinson, E. , Hydrocolloids at interfaces and the influence on the properties of dispersed systems. Food Hydrocoll. 2003, 17, 25–39.

